# ASS1 inhibits triple-negative breast cancer by regulating PHGDH stability and de novo serine synthesis

**DOI:** 10.1038/s41419-024-06672-z

**Published:** 2024-05-06

**Authors:** Wensong Luo, Zizheng Zou, Yuan Nie, Junli Luo, Zhengnan Ming, Xiyuan Hu, Tiao Luo, Min Ouyang, Mingquan Liu, Huicheng Tang, Yuanzhu Xie, Kunjian Peng, Ling Chen, Jiang Zhou, Zhiyong Luo

**Affiliations:** 1 Department of Biochemistry and Molecular Biology, Hunan Province Key Laboratory of Basic and Applied Hematology, Hunan Key Laboratory of Animal Models for Human Diseases, School of Life Sciences, Xiangya School of Medicine, Central South University, Changsha, China; 2 Yiyang Key Laboratory of Chemical Small Molecule Anti-Tumor Targeted Therapy, Department of Scientific Research, Yiyang Medical College, Yiyang, 413000 China; 3 The Cancer Research Institute, Hengyang Medical School, University of South China, Hengyang, China; 4 Hunan Key Laboratory of Oral Health Research & Xiangya Stomatological Hospital & Xiangya School of Stomatology, Central South University, Changsha, China; 5 Department of Pharmacy, The Second Xiangya Hospital of Central South University, Changsha, China

**Keywords:** Cancer, Breast cancer

## Abstract

Argininosuccinate synthase (ASS1), a critical enzyme in the urea cycle, acts as a tumor suppressor in many cancers. To date, the anticancer mechanism of ASS1 has not been fully elucidated. Here, we found that phosphoglycerate dehydrogenase (PHGDH), a key rate-limiting enzyme in serine synthesis, is a pivotal protein that interacts with ASS1. Our results showed that ASS1 directly binds to PHGDH and promotes its ubiquitination-mediated degradation to inhibit serine synthesis, consequently suppressing tumorigenesis. Importantly, the tumor suppressive effects of ASS1 were strongly abrogated by PHGDH knockout. In addition, ASS1 knockout and knockdown partially rescued cell proliferation when serine and glycine were depleted, while the inhibitory effect of ASS1 overexpression on cell proliferation was restored by the addition of serine and glycine. These findings unveil a novel role of ASS1 and suggest that the ASS1/PHGDH serine synthesis pathway is a promising target for cancer therapy.

## Introduction

Breast cancer, one of the most common causes of death of women, is a variable disease at the molecular level [1–3]. Among the types of this disease, triple-negative breast cancer (TNBC) is the most challenging to address, and is characterized by a high degree of malignancy, poor prognosis, high recurrence rate and easy development of distant metastases [4]. Although mastectomy and chemotherapy significantly enhanced the survival of patients with triple-negative breast cancer, prevention and treatment will always be impeded without a full understanding of the potential underlying mechanisms and pathogenesis involved [2]. Therefore, further comprehensive and in-depth research into the mechanisms underlying the occurrence and development of this disease is urgently needed to identify new therapeutic targets and thereby increase the therapeutic efficacy.

ASS1 is a recognized rate-limiting enzyme in arginine synthesis that catalyzes the production of argininosuccinate. A lack of ASS1 expression or ASS1 downregulation is very common in cancer, including myxofibrosarcomas, breast cancer, malignant melanoma, liver cancer, prostate cancer, pancreatic cancer, renal cancer, and osteosarcomas [5–8]. Several reports have shown that ASS1 is underexpressed in a range of tumors and functions as a tumor suppressor [5–13]. Furthermore, ASS1 is associated with chemoresistance, invasion, recurrence, and poor clinical outcomes, including breast cancer, HCC, renal cell carcinoma, and prostate cancer [10, 13, 14], in which ASS1 expression is lost [8, 10, 11]. Our previous work showed that increased activity of ASS1 induced by its activator exerts an anticancer function by reducing pyrimidine synthesis. However, the addition of pyrimidines only partially rescued cell viability, suggesting that there may be another antitumor pathway involving ASS1 [13]. Furthermore, the molecular pathways that are modulated by ASS1 are still not fully exhaustive, and its function in TNBC is even less well understood.

Cell metabolic reprogramming is a well-recognized hallmark of cancer that exploits metabolites for rapid growth and proliferation [15, 16]. An important metabolic adaptation to the rapid proliferation of many human cancers is the increased biosynthesis of serine and glycine [17, 18]. Serine and glycine are nonessential amino acids. However, these molecules are involved in many metabolic processes that are critical for cancer cell growth and proliferation, which involve the synthesis of proteins, amino acids and glutathione, and can act as essential CO donors for the folate cycle, facilitating nucleotide synthesis, methylation reactions and the production of NADPH [19–23]. Two sources of serine, extracellular import via transporters and de novo synthesis via the serine biosynthetic pathway (SSP) [19, 20], are required by cells. PHGDH catalyzes the committed step in de novo serine biosynthesis [24, 25]. PHGDH, the first and only rate-limiting enzyme in serine synthesis, generates 3-phosphohydroxypyruvate (3-PPyr) through the coupled oxidation of 3-PG by NAD^+^ [23, 24, 26]. Recent reports in tumors have shown that PHGDH is highly expressed in breast cancer, melanoma, non-small cell lung cancer, hepatocellular carcinoma, pancreatic cancer, glioma, and gastric cancer tissues compared to normal tissues [24, 26–34] and that PHGDH overexpression accelerates cancer progression and promotes drug resistance by activating serine synthesis [35–37]. Targeting PHGDH as a treatment strategy for cancers with PHGDH overexpression may strongly inhibit cancer progression, indicating that high expression of PHGDH in tumors is a critical therapeutic target. Several studies of breast cancer and melanoma have shown that gene amplification contributes to PHGDH overexpression [24, 38] and that PHGDH expression can be upregulated by the transcription factor NRF2 in non-small cell lung cancer [29]. In addition, PHGDH can be ubiquitinated by Parkin in breast cancer, thus low Parkin expression contributes to PHGDH overexpression [39]. However, the regulatory role of PHGDH in cancer has not been fully elucidated.

Here, we identified PHGDH as an important protein that interacts with ASS1 and found that ASS1 promotes the ubiquitination-mediated degradation of PHGDH to inhibit serine synthesis and TNBC cell proliferation, which significantly contributes to the anticancer of ASS1. ASS1 knockout and knockdown resulted in increased serine synthesis as well as tumor growth through stabilization of PHGDH protein levels, and this effect was significantly abrogated by PHGDH knockout. These findings demonstrate a crucial mechanism that underlies the anticancer properties of ASS1 and the regulation of PHGDH in TNBC.

## Materials and methods

### Cell culture

All cell lines were acquired through the American Type Culture Collection (ATCC, Manassas, VA, USA) and identified within the last 3 years by short tandem repeat (STR) analysis. BT549, BT474, HCC1500, HCC1806, HCC1937, MDA-MB-231, and T47D cells were grown in RPMI 1640. Hs578T, MCF-7, SKBR3, and BT20 cells were grown in DMEM. MDA-MB-468 and 293 T cells were grown in DMEM supplemented with high glucose. All media were supplemented with 10% FBS and 1% penicillin‒streptomycin. MCF10A cells were grown in DMEM/F12 supplemented with 5% horse serum, 1% penicillin‒streptomycin and other materials as described in this study [13]. All the cells were grown in a 37 °C and 5% CO2 humidified incubator.

### Mouse xenograft tumor models

Four-week-old female BALB/c nu/nu mice were obtained from Hunan SJA Laboratory Animal company (Hunan, china), and reared in a specific pathogen-free environment. Protocols for animal experiments were conducted in accordance with the Experimental Animal Ethics Committee of Hunan Normal University (Changsha, China), No. D2022042. Nude mice were randomly divided into four groups (*n* = 6 per group). For xenograft experiments, Con MDA-MB-468 cells, MDA-MB-468 cells with ASS1 knockout (KO), PHGDH KO, and ASS1 and PHGDH double KO (5 × 10^6^) were injected subcutaneously into BALB/c nu/nu mice. Tumor volumes were measured every 7 days using calipers and calculated with L × W^2^ × 0.52, L: length, W: width.

### Cell viability experiments

Cell viability was measured by methyl thiazolyl tetrazolium (MTT) and colony formation experiments. Briefly, equal amounts of cells were plated in 96-well plates (0 h) or 6-well plates and cultured for 96 h or 12 days respectively. At the specified time points, the cells were incubated with MTT (5 mg/mL, 20 µL) for 4 h in 96-well plates, then DMSO was added and lysed for 10 min. The absorbance value (OD) was measured at 490 nm with a Multiskan Go microplate reader (Thermo Scientific, USA). For colony formation, 4% paraformaldehyde was added for fixation, the cells were stained with 0.1% crystal violet, and washed with PBS.

### GST pulldown assays

The coding sequences (CDSs) of ASS1, PHGDH and their truncated forms were cloned into the PGX-4T-1 and PET-28a vectors respectively using the ClonExpress® II One Step Cloning Kit (Vazyme, catalog C112-01). Transformation of the GST-ASS1, GST-PHGDH, His-PHGDH and His-ASS1 plasmids into *E. coli* (strain BL21 DE3) was performed. Induction of prokaryotic protein expression was performed with 0.5 mM IPTG at 16 °C overnight. The purified GST-ASS1/PHGDH protein was immobilized on MagneGST^TM^ Glutathione Particles (Promega, USA), and the purified His-PHGDH/ASS1 protein was subsequently added overnight at 4 °C with slow rotation, after which the mixture was washed with PBST and analyzed by western blot. The GST protein was used as a negative control.

### Real-time qPCR assays

Total RNA was isolated from the cells using TRIzol^®^ (catalog 15596026, Invitrogen) and reverse transcribed to cDNA using a Vazyme Reverse Transcription Kit (catalog R212-01 Vazyme). The ChamQ Universal SYBR qPCR Master Mix was employed for performing real-time PCR (catalog Q711-03, Vazyme). GAPDH expression was used for normalization of the results. The primers used for qRT‒PCR are shown in Supplementary Table 1.

### In vivo ubiquitination assays

Myc-ASS1, 3 × Flag-PHGDH, and HA-Ub plasmids were transfected into 293 T cells for 72 hours. MDA-MB-468 cells with or without ASS1 knockout were transfected with 3 × Flag-PHGDH and HA-Ub expression vectors for 72 hours. All the cells were treated with MG132 (catalog S2619, Selleck) (10 μM) for 8 hours before collection. Ubiquitination of 3 × Flag-PHGDH was performed by an IP assay of 3 × Flag-PHGDH using an anti-Flag antibody followed by a western blot analysis with an anti-HA antibody. The antibodies used in this study are listed in Supplementary Table 2.

### LC‒MS/MS analysis of total metabolites

Equal amounts of cells were collected and washed with PBS. Equal amounts of a mixture of methanol, acetonitrile and water (4:4:2) were added, sonicated, incubated for 30 min on ice, dried, dissolved in 200 μL of the mobile phase (99% ammonium formate −1% acetonitrile) and automatically sampled in 1 µL for detection. The data processing and instrument parameter information used are provided in the Supplementary Materials.

### GC‒MS measurement of ^13^C-labeled metabolites

The same amounts of cells were plated (5 × 10^6^ cells) and cultured overnight. The old solution was removed, and the cells were washed three times with PBS. The cells were cultured in DMEM supplemented with 10% dialyzed serum and 25 mM U-^13^C_6_ glucose (catalog IR-24093, isoreag) for 4 h. Metabolites were extracted using a 50% aqueous methanol mixture, immersed in liquid nitrogen for 30 min, and then thawed on ice. Next, 400 µL of chloroform was added, and the mixture was vortexed for 60 s and subsequently centrifuged at 14,000 rpm (4 °C) for 20 min. The supernatant was subsequently transferred, evaporated and stored at −80 °C before analysis. Metabolites were derivatized before GC/MS analysis, 70 μl of O-isobutylhydroxylamine hydrochloride was added, and the mixture was incubated for 20 min at 85 °C. After the samples reached room temperature, 30 µl of *N-tert*-butyldimethylsilyl-*N*-methyltrifluoroacetamide (MTBSTFA) was added, and the samples were reincubated for 60 min at 85 °C. Finally, the samples were centrifuged for 20 min at 14,000 rpm (4 °C). The supernatant was transferred to an autosampler vial for GC/MS analysis. The data analysis and instrument parameter information are provided in the Supplementary Materials.

### Analysis of the protein half-life

Cycloheximide (catalog S7418, Selleck) or DMSO was added to the cells for the indicated durations (0–24 h). After lysis, western blot analysis was carried out using an anti-PHGDH antibody.

### Western blotting

Cells or xenograft tumors were lysed using RIPA (catalog V900854-100ML, VETEC) with 1 mM PMSF, and protease inhibitors (catalog S8830, Sigma‒Aldrich). Protein concentrations were quantified using the BCA protein test package (catalog P0010, Beyotime). After the proteins were separated, they were transferred to a PVDF membrane (catalog IPVH00010, Millipore), blocked with 5% nonfat milk, and incubated with the corresponding antibodies. On the second day, the bands were incubated with the corresponding secondary antibodies. HRP chemiluminescent substrates were used to visualize the immunoreactive bands (catalog BMU102-CN, Abbkine). The bands were quantified from the western blot results using Image Lab. The antibodies used in this study are listed in Supplementary Table 2.

### CRISPR/Cas9 for knockout

The guide RNAs (gRNAs) were generated by the CRISPR online service (http://crispor.tefor.net/). The generation of pLentiCRISPR was conducted in accordance with the Zhang Lab’s protocol. The ASS1 and PHGDH gRNAs were cloned into pLentiCRISPR v2 respectively and resulted in lentiviruses. For generation of stable ASS1 knockout and PHGDH knockout cells, the cells were infected with lentivirus and selected with a high concentration of puromycin for 2 days, after which monoclonal cells were generated via a colony formation assay. The knockout effect of the cloning of the single cells was validated by western blot and DNA sequencing. The primers used for sgRNA are listed in Supplementary Table 3.

### ASS1 overexpression and knockdown and PHGDH knockdown

The coding sequence (CDS) of ASS1 and the short hairpin RNAs (shRNAs) of ASS1 and PHGDH were subcloned into the Phage-N-Flag-Puro and pLKO.1 vectors, respectively, and packaged into lentiviruses to infect cells. Then, the cells were selected with puromycin. The shRNA sequences of ASS1 and PHGDH are shown in Supplementary Table 4.

The CDS of human ASS1 was subcloned into the Phage-N-Flag-Puro lentiviral vector.

### Coimmunoprecipitation

Cells were lysed with IP lysis solution (catalog P0013, Beyotime), anti-ASS1 and anti-PHGDH antibodies were added respectively, and the mixture was subsequently incubated at 4 °C with slow rotation. Protein A/G magnetic beads (catalog XE351456, Thermo Scientific) were added for 3 h with slow rotation, washed with IP lysis, and then detected by western blot. The negative control was rabbit IgG, and the antibodies for this study are listed in Supplementary Table 2.

### LC‒MS/MS analysis

Co-IP and LC‒MS/MS experiments were used to determine potential ASS1 interacting proteins. Briefly, 293 T cells were infected with p3XFlag-ASS1-cmv-10 and the empty vector for 48 h. ASS1-3xFlag and 3xFlag were pulled down using an anti-Flag affinity gel (catalog B23101, Bimake), followed by SDS‒PAGE electrophoresis after washing. Silver staining was performed and specific adhesive strips were cut for LC‒MS/MS analysis at the BGI.

### Immunofluorescence

MDA-MB-468 cells were cultured in 12-well plates with cell crawling film and grown to 70% coverage. After a wash, 4% paraformaldehyde and water at 80 °C were used for fixation and antigen retrieval, respectively. Slides were subsequently blocked with 5% BSA (with 0.3% Triton-X100) and incubated with primary antibodies over 12 h at 4 °C. After a wash, fluorescent secondary antibody was added. DAPI was stained for 5 min and washed with PBS. Slides were sealed with an antifluorescence quenching sealer, and a drop of neutral gum was placed around the slide. The samples were subsequently stored in the dark, after which images were taken immediately. The antibodies used in this study are shown in Supplementary Table 2.

### Clinical samples

All TNBC tissues were obtained from the Second Xiangya Hospital of Central South University (Changsha, China). Informed consents were obtained from all participants in accordance with the Declaration of Helsinki. All protocols using human specimens were approved by the Institutional Review Board of the Second Xiangya Hospital of Central South University.

### Immunohistochemistry (IHC)

Human breast tumor tissues were provided by the Second Xiangya Hospital, Central South University (Changsha, China). Tissue fixed in formalin, embedded in paraffin and cut into 6 µm thick sections. The sections were deparaffinized and rehydrated. Boiled citrate buffer was used for antigen retrieval. Peroxidase closure was performed using 3% H_2_O_2_ with 0.3% Triton. Slides were blocked with 5% BSA and then incubated with the corresponding antibodies over 12 hours at 4 °C. After a wash, a universal two-step assay kit was used to add liquids A and B (Mouse/Rabbit Enhanced Polymer Assay System) (catalog PV-9000, ZSGB -BIO). DAB working solution was added. Hematoxylin staining was conducted, and then washed with PBS. The sections were then incubated in hydrochloric acid alcohol (0.5%) for 2 s, washed with PBS, lithium carbonate returned to blue, and then placed in a slow stream for 20 min. Alcohol gradient dehydration, xylene clearing, and immediate blocking of the film were performed. The IHC scoring method is provided in the Supplementary Materials, and the antibodies used in this study are listed in Supplementary Table 2.

### Statistics

All the data are expressed as the means ± Standard Deviation (SD) from at least three independent experiments, as indicated in the figure legends. Statistical significance testing was conducted by *T*-test, one-way, or two-way ANOVA. Statistical analysis was conducted with GraphPad Prism 8. *p* < 0.05 was considered as statistically significant (*p < 0.05; **p < 0.01; ***p < 0.001; n.s., no significance).

## Results

### PHGDH interacts directly with ASS1

**Fig. 1:**
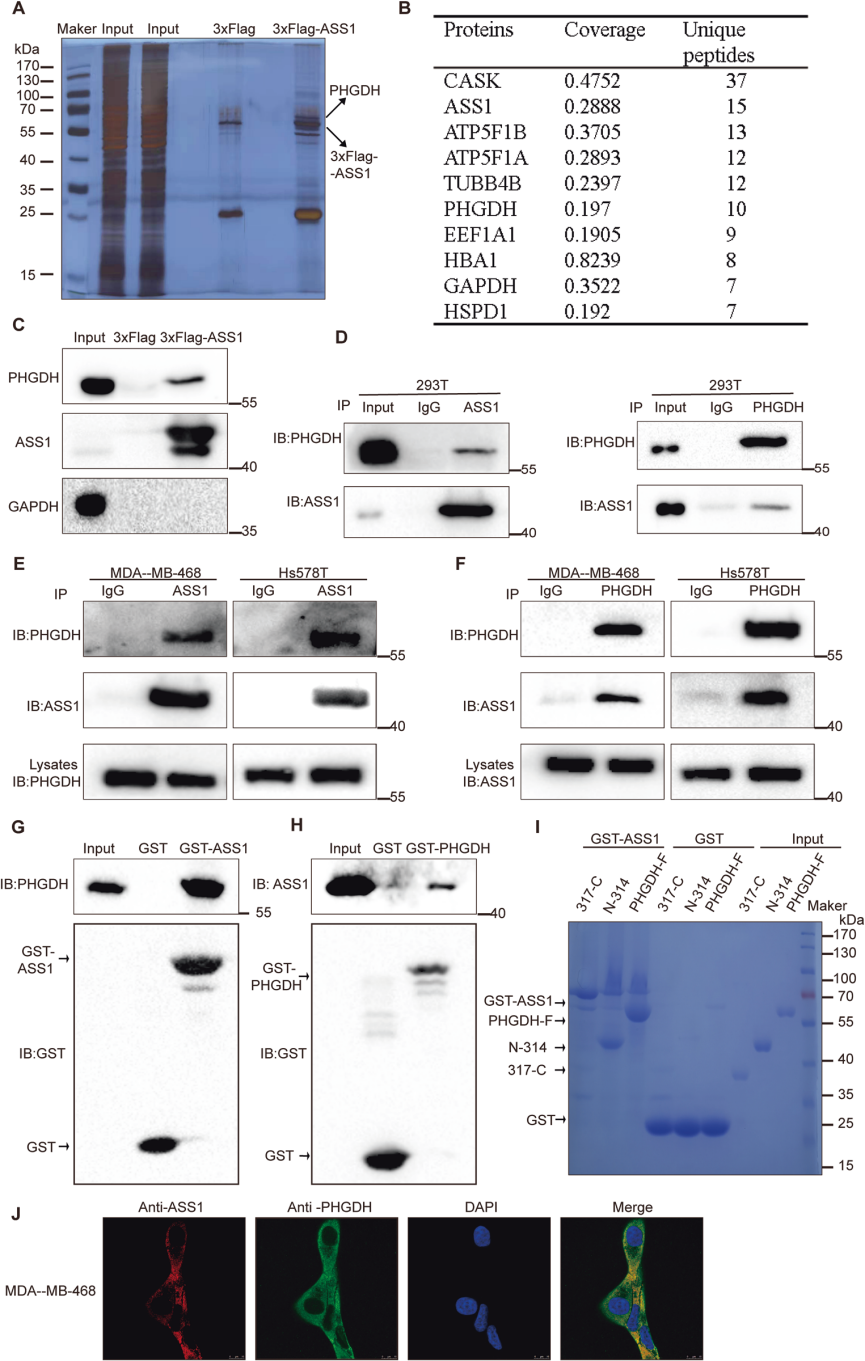
PHGDH directly interacts with ASS1. **A** Silver staining results of 293 T cells stably expressing the empty vector 3xFlag or 3xFlag-ASS1 that were subjected to co-IP assays using anti-Flag affinity gel beads. **B** The top ten ASS1-interacting proteins according to the number of identified unique peptides. **C** Validation of the mass spectrometry results of 293 T cells stably expressing the empty vector 3xFlag or 3xFlag-ASS1 by immunoblotting. **D** Co-IP and reciprocal co-IP assays of ASS1 and PHGDH in293T cells. Cell lysates were subjected to IP with the indicated antibodies and analyzed via IB. Normal rabbit IgG was used as a negative control. **E**, **F** Co-IP and reciprocal co-IP assays of ASS1 and PHGDH in MDA-MB-468 and Hs578 cells. Cell lysates were subjected to IP with the indicated antibodies and analyzed via IB. Normal rabbit IgG was used as a negative control. **G**, **H** Direct interaction of the recombinant GST-ASS1 and His-PHGDH proteins (**G**) or the recombinant GST-PHGDH and His-ASS1 proteins (**H**) analyzed by in vitro GST pull-down assays. The proteins were pulled down with the indicated recombinant protein and analyzed via IB. GST was used as a negative control. **I** Full-length PHGDH (PHGDH-F) and its truncated forms (N-314 and 317-C) with recombinant GST-ASS1 for the GST pull-down assay. GST was used as a negative control. **J** Immunofluorescence images showing the colocalization of ASS1 (red) and PHGDH (green) in MDA-MB-468 cells. The nuclei were counterstained with DAPI (blue). Scale bars: 10 μm.

Our previous study showed that the activator of ASS1 increases its activity and thus exerts anticancer effects by reducing pyrimidine synthesis. However, pyrimidine supplementation only partially restored cell viability, suggesting that ASS1 has an additional antitumor mechanism. To further reveal the mechanism of the tumor suppressor function of ASS1 in TNBC, we conducted coimmunoprecipitation assays followed by LC‒MS/MS analysis in 293 T cells to identify potential proteins that interact with ASS1. Silver staining (Fig. 1A) and LC‒MS/MS revealed that PHGDH is the most potential binding protein of ASS1 (Fig. 1B), and these results were confirmed by western blotting (Fig. 1C). Co-IP followed by western blot assays in 293 T cells demonstrated the interaction between endogenous ASS1 and PHGDH (Fig. 1D). Furthermore, this interaction was observed in MDA-MB-468 and HS578T cells (Fig. 1E, F). To investigate whether ASS1 interacts directly with PHGDH, we performed GST pull-down experiments in vitro. His-PHGDH and His-ASS1 proteins were prokaryotically expressed and purified (Fig. S1). We identified a direct interaction between GST-ASS1 and His-PHGDH (Fig. 1G). Additionally, the same results were observed between GST-PHGDH and His-ASS1 (Fig. 1H). We further sought to identify the region of PHGDH that interacted with ASS1 by expressing two truncated forms of PHGDH (the catalytic and regulatory domains) and found that the amino acid N-314(catalytic domain) directly bound to ASS1 (Fig. 1I). Furthermore, 6 of the 10 specific peptides of PHGDH identified by LC-MS/MS were located on amino acid N-314 (Fig. S2). In addition, we performed immunofluorescence experiments, which showed substantial colocalization of the two proteins (Fig. 1J). Taken together, these results show that ASS1 interacts directly with PHGDH in TNBC cells.

### PHGDH protein levels are downregulated by ASS1 and inversely correlated with ASS1 in TNBC tissue

**Fig. 2:**
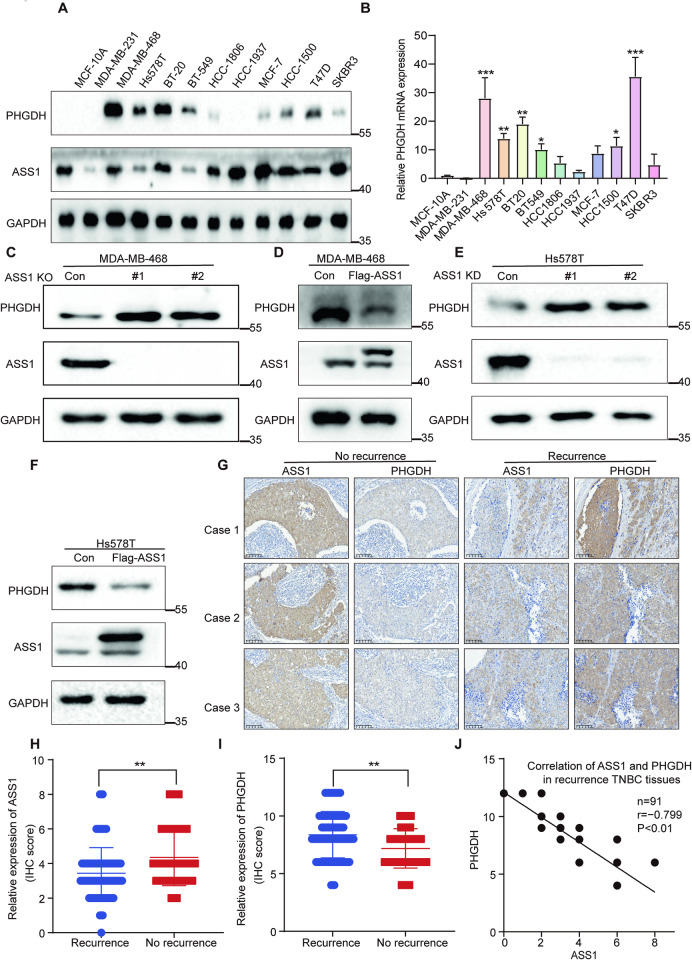
ASS1 expression is inversely correlated with PHGDH protein expression in TNBC. **A** Immunoblotting (IB) of PHGDH and ASS1 protein in MCF-10A and 11 breast cancer cell lines, with GAPDH serving as a loading control. **B** RT-qPCR analysis of PHGDH mRNA levels in 11 breast cancer cell lines relative to the MCF-10A cell line. GAPDH was used as an internal control. Three independent experiments were performed, and the data are shown as the mean ± SD with *p*-values based on one-way ANOVA (*n* = 3, *p < 0.05, **p < 0.01, ***p < 0.001, n.s., not significant). **C**, **D** Immunoblotting assays assessing the expression levels of PHGDH in MDA-MB-468 cells with ASS1 knockout (**C**) or ASS1 overexpression (**D**). **E**, **F** Immunoblotting assays assessing the expression levels of PHGDH in Hs578T cells with ASS1 knockdown (**E**) or overexpression (**F**). **G** Analysis of ASS1 and PHGDH protein levels in 91 tissues from recurrent TNBC patients and 70 tissues from nonrecurrent TNBC patients by IHC assays. The images shown are from three representative experiments. Scale bars: 200 μm. H-I ASS1 (**H**) and PHGDH (**I**) protein expression levels in recurrent (*n* = 91) and no recurrence (*n* = 70) human TNBC patients. Data are shown as mean ± SD with *p-*values based on unpaired *T*-test, **p < 0.01. **J** Correlation analysis of ASS1 and PHGDH protein expression in human recurrent triple-negative breast cancer tissues (*n* = 91) revealed a significant correlation (Pearson’s correlation, *r* = −0.799; *p* < 0.01).

PHGDH is often overexpressed in breast cancer [24]. Thus, the mRNA and protein levels of PHGDH were measured. The results showed higher PHGDH protein (Fig. 2A) and mRNA (Fig. 2B) expression in most breast cancer cells than in human normal mammary epithelial cells. Given that PHGDH interacts with ASS1, we knocked out ASS1 in MDA-MB-468 cells with high levels of PHGDH. Unexpectedly, we found that ASS1 knockout upregulated PHGDH protein expression (Fig. 2C). Conversely, ASS1 overexpression downregulated PHGDH protein expression (Fig. 2D). Similar results were also shown in Hs578T cells with high levels of PHGDH (Fig. 2E, F). However, PHGDH knockout or knockdown (Fig. S3A, B) had little effect on ASS1 protein levels. Furthermore, real-time qPCR indicated that ASS1 did not alter the mRNA level of PHGDH in cells (Fig. S4A–D). In addition, the IHC results showed that ASS1 was negatively associated with breast cancer recurrence (Fig. 2G, H), whereas PHGDH was positively associated with breast cancer recurrence (Fig. 2G, I), and ASS1 was negatively associated with PHGDH in TNBC patient tissue (Fig. 2J, S5A). Taken together, these results indicate that PHGDH may be associated with TNBC recurrence, that its protein expression is downregulated by ASS1, and it is inversely associated with ASS1 expression in TNBC tumor tissue.

### ASS1 promotes PHGDH degradation mainly through the ubiquitin‒proteasome pathway

**Fig. 3:**
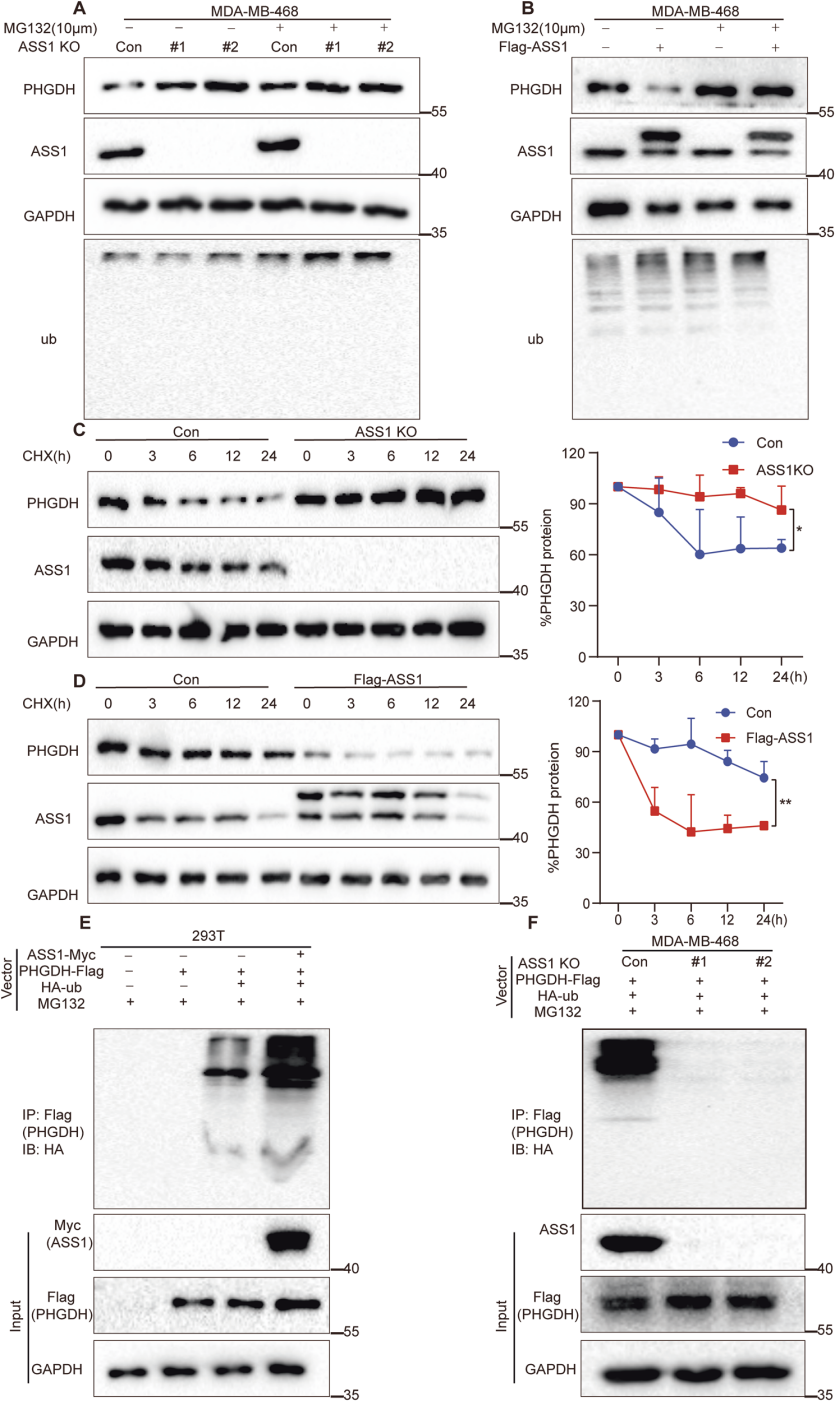
ASS1 promotes PHGDH degradation by ubiquitination. **A**, **B** Immunoblotting assays examining the expression levels of PHGDH in MDA-MB-468 cells with ASS1 knockout (**A**) or ASS1 overexpression (**B**) treated with DMSO or 10 μM MG132. Ub was used as a positive control. **C**, **D** Immunoblotting assays assessing the expression levels of PHGDH in MDA-MB-468 cells with ASS1 knockout (**C**) or ASS1 overexpression (**D**) treated with DMSO or CHX (200 μM). GAPDH was used as a loading control. For each cell, the quantification of the relative endogenous PHGDH protein levels from three independent experiments is shown. Data are means ± SD with *p*-values based on unpaired *T*-tests (*p < 0.05). **E** Immunoprecipitation (IP)-IB of ubiquitinated PHGDH using 293 T cells transfected with ASS1-Myc, PHGDH-Flag, or HA-Ub and treated with MG132 (10 μM) for 8 h before harvest. **F** Immunoprecipitation (IP)-IB of ubiquitinated PHGDH using MDA-MB-468 Con or ASS1 knockout cells transfected with PHGDH-Flag, or HA-Ub and treated with MG132 (10 μM) for 8 h before harvest.

Two main pathways, the proteasomal and lysosomal pathways, are involved in protein degradation [40–42]. Bafilomycin A1 (BafA1) was used to explore lysosomal degradation and MG-132 was used to explore the proteasomal pathway. The efficacy of BafA1 in MDA-MB-468 cells was confirmed by a decrease in the protein level of LC3B, a known target of the lysosome. However, the protein levels of PHGDH were unaltered in both ASS1 knockout and ASS1-overexpressing cells, suggesting little turnover of PHGDH by lysosomes in cells (Fig. S6A, B). The effectiveness of MG132 was confirmed by an increase in ubiquitinated proteins. We found that ASS1 knockout increased PHGDH protein levels, but MG132 treatment abrogated the promoting effect of ASS1 knockout (Fig. 3A). ASS1 overexpression decreased PHGDH protein levels, however, MG132 treatment abrogated the inhibitory effect of ASS1 overexpression (Fig. 3B). Together, these results indicate that ASS1 may downregulate PHGDH in cells mainly through ubiquitin-proteasomal degradation.

We then investigated whether ASS1 affects the stability of the PHGDH protein by measuring its half-life in cells. The protein synthesis inhibitor CHX was used to treat MDA-MB-468 cells with or without ASS1 gene knockout for different durations. Relative to the control cells, ASS1 knockout significantly delayed the half-life of PHGDH (Fig. 3C). Conversely, the overexpression of ASS1 reduced the half-life of the PHGDH protein (Fig. 3D). To determine whether ASS1 affects PHGDH ubiquitination, in vivo ubiquitination experiments were conducted in 293 T cells. The results indicated that ASS1-Myc significantly enhanced the ubiquitination of PHGDH-Flag (Fig. 3E). Moreover, ASS1 knockout substantially reduced PHGDH-Flag ubiquitination in MDA-MB-468 cells (Fig. 3F). Taken together, these results show that ASS1 may downregulate PHGDH through ubiquitination-mediated and proteasomal degradation.

### ASS1 inhibits serine synthesis through PHGDH

**Fig. 4:**
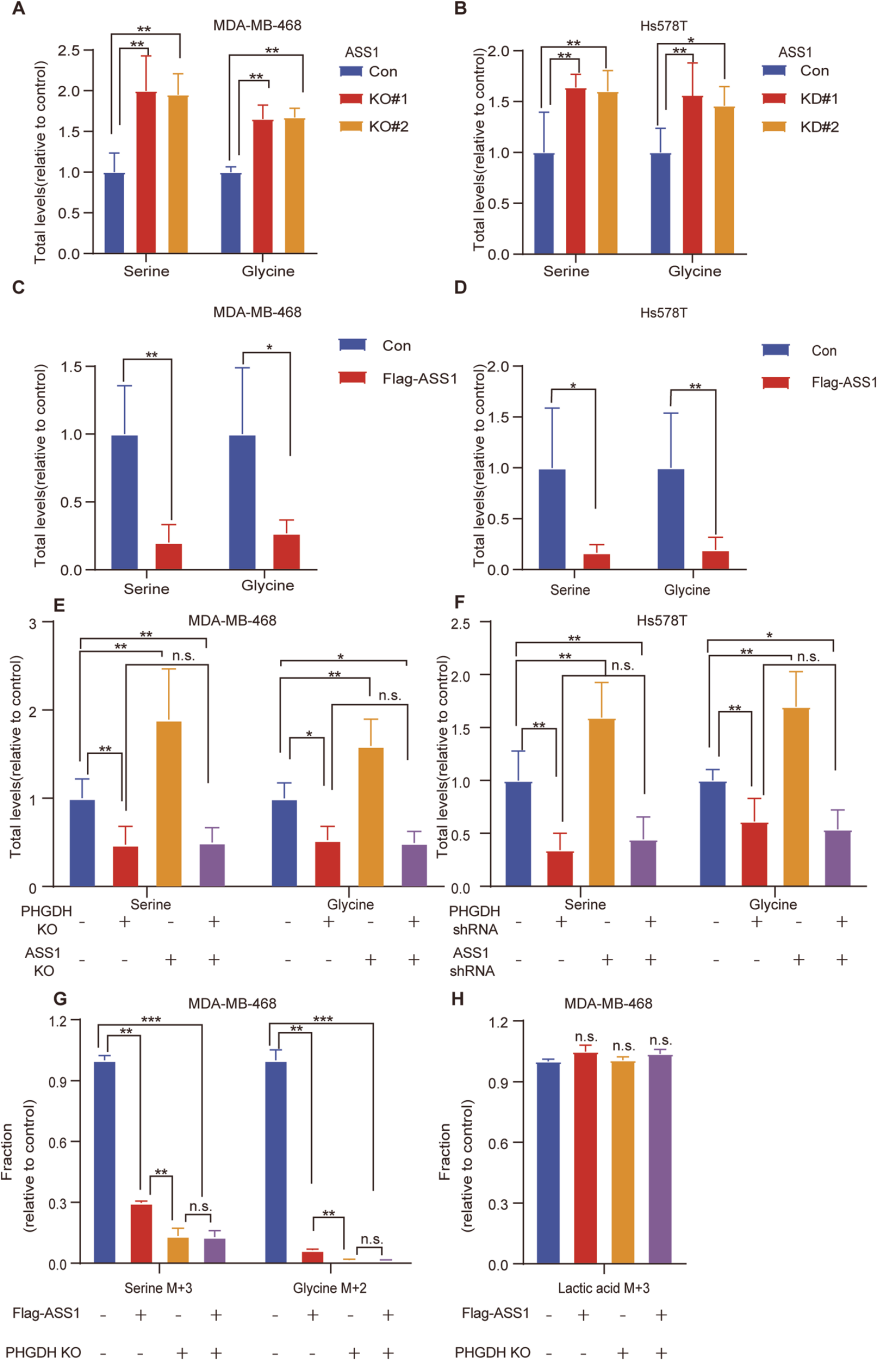
ASS1 inhibits PHGDH-mediated de novo serine synthesis. **A** MDA-MB-468 cells with ASS1 knockout via CRISPR/Cas9 (KO#1 and KO#2) were subjected to LC‒MS/MS to determine the concentrations of serine and glycine. Representative images of the relative metabolic results are shown in **A**. Six independent experiments were performed and data are the means ± SD with *p-*values based on unpaired *T*-test. (*n* = 6, **p < 0.01). **B** Hs578T cells with ASS1 knockdown by shRNA (KD#1 and KD#2) were subjected to LC‒MS/MS to determine the concentrations of serine and glycine. Representative images of the relative metabolic results are shown in **B**. Six independent experiments were performed and data are the means ± SD with *p-*values based on unpaired *T*-test. (*n* = 6, *p < 0.05, **p < 0.01). **C**, **D** MDA-MB-468 and Hs578T cells stably expressing the control empty plasmid or Flag-ASS1 were subjected to LC‒MS/MS to determine the concentrations of serine and glycine. Representative images of the relative metabolic results are shown in **C** and **D**. Six independent experiments were performed and data are the means ± SD with *p*-values based on unpaired *T*-test. (*n* = 6, *p < 0.05, **p < 0.01). **E** PHGDH, ASS1, PHGDH and ASS1 were knocked out by CRISPR/Cas9 in MDA-MB-468 cells, and the cells were subjected to LC‒MS/MS to determine the concentrations of serine and glycine. Representative images of the relative metabolic results are shown in (**E**). Six independent experiments were performed and data are the means ± SD with *p*-values based on two-way ANOVA. (*n* = 6, *p < 0.05, **p < 0.01, n.s., not significant). **F** Hs578T cells with PHGDH, ASS1, PHGDH and ASS1 knocked down by shRNA were subjected to LC‒MS/MS to determine the concentrations of serine and glycine. Representative images of the relative metabolic results are shown in F. Six independent experiments were performed and data are the means ± SD with *p*-values based on two-way ANOVA. (*n* = 6, *p < 0.05, **p < 0.01, n.s., not significant). **G** MDA-MB-468 cells cultured in medium containing 25 mM [U-13C] D-glucose with 0.4 mM serine/glycine were lysed for stable isotope flux analysis. Flag-ASS1 decreased the incorporation of ^13^ C into serine and glycine in MDA-MB-468 cells, which was largely abolished by PHGDH knockout. Three independent experiments were performed and data are the means ± SD with *p*-values based on two-way ANOVA (*n* = 3, **p < 0.01, ***p < 0.001, n.s., not significant). **H** MDA-MB-468 cells cultured in medium containing 25 mM [U-13C] D-glucose with 0.4 mM serine/glycine were lysed for stable isotope flux analysis. Neither Flag-ASS1, PHGDH knockout, nor Flag-ASS1 and PHGDH knockout significantly affected the incorporation of ^13^ C into lactic acid in MDA-MB-468 cells. Three independent experiments were performed and data are the means ± SD with *p*-values based on one-way ANOVA. (*n* = 3, n.s., not significant).

PHGDH is a pivotal enzyme in the serine synthesis pathway, which produces serine and glycine to facilitate the synthesis of amino acids, lipids, nucleotides and glutathione [19, 21]. Since ASS1 can ubiquitinate and degrade PHGDH, we speculate that ASS1 may affect serine synthesis. To investigate serine synthesis, cells were maintained in serine- and glycine-deprived media supplemented with dialyzed serum. Compared with control cells, ASS1 knockout and knockdown significantly increased serine and glycine levels in MDA-MB-468 (Fig. 4A) and Hs578T cells (Fig. 4B). Conversely, ASS1 overexpression significantly decreased serine and glycine levels (Fig. 4C, D). We then investigated whether ASS1 inhibits serine synthesis by downregulating PHGDH. PHGDH knockout and knockdown significantly decreased serine and glycine levels (Fig. 4E, F), which was consistent with the results of previous reports [39]. Interestingly, both PHGDH knockout and knockdown significantly abrogated the promoting effect of ASS1 knockout and knockdown in both cell lines (Fig. 4E, F). Similarly, PHGDH knockdown or knockout largely abrogated the inhibitory effect of ASS1 overexpression in both cell lines (Fig. S7A, B). Importantly, to further determine newly synthesized serine and glycine, we incubated MDA-MB-468 cells in medium containing[U-^13^C_6_]-glucose for stable isotope tracing assays. GC-MS-based metabolomic analysis showed that Flag-ASS1 expression inhibited newly synthesized serine (with a mass shift of 3 [M + 3]), and glycine (with a mass shift of 3 [M + 2]) (Fig. 4G), which could be abolished by PHGDH knockout, but had no effect on lactic acid (with a mass shift of 3 [M + 3]) (Fig. 4H), pyruvic acid and 3-phosphoglycerate (with a mass shift of 3 [M + 3]) (Fig. S4C, D). Together, these results demonstrated that ASS1 inhibits serine synthesis by downregulating PHGDH in cells.

### ASS1 inhibits TNBC growth by downregulating PHGDH

**Fig. 5:**
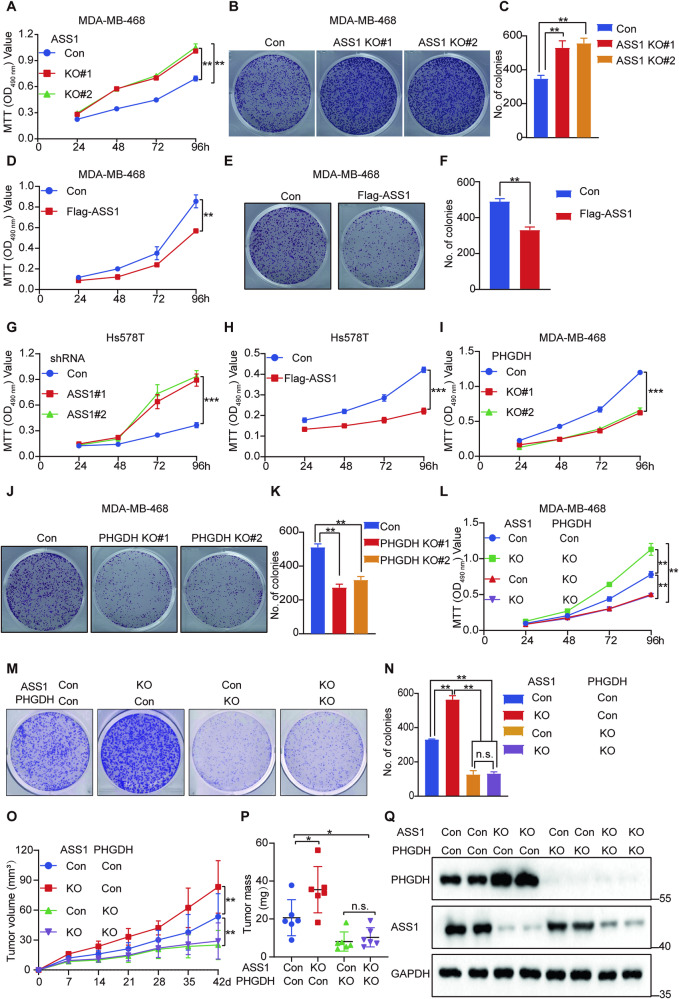
ASS1 inhibits TNBC growth by regulating PHGDH stability. **A** MDA-MB-468 cells with ASS1 knockout (KO#1 and KO#2) via CRISPR/Cas9 were subjected to MTT assays. Four independent experiments were performed and data are shown as the means ± SD with *p*-value based on unpaired *T*-test (*n* = 4, **p < 0.01). **B**, **C** MDA-MB-468 cells with ASS1 knockout (KO#1 and KO#2) via CRISPR/Cas9 were subjected to colony formation assays. Representative images and quantitative results of colonies are shown. Three independent experiments were performed and data are shown as the means ± SD with *p-*value based on unpaired *T*-test (*n* = 3, **p < 0.01). **D** MDA-MB-468 cells stably expressing the control empty plasmid or Flag-ASS1 were subjected to MTT assays. Four independent experiments were performed and data are shown as the means ± SD with *p*-value based on unpaired *T*-test (*n* = 4, **p < 0.01). **E**, **F** MDA-MB-468 cells stably expressing the control empty plasmid or Flag-ASS1 were subjected to colony formation assays. Representative images and quantitative results of colonies are shown. Three independent experiments were performed and data are shown as the means ± SD with *p*-value based on unpaired *T*-test (*n* = 3, **p < 0.01). **G** Hs578T cells with ASS1 knockdown by shRNA (KD#1 and KD#2) were subjected to MTT assays. Four independent experiments were performed and data are shown as the means ± SD with *p*-value based on unpaired *T*-test (*n* = 4, ***p < 0.001). **H** Hs578T cells stably expressing the control empty plasmid or Flag-ASS1 were subjected to MTT assays. Four independent experiments were performed and data are shown as the means ± SD with *p*-value based on unpaired *T*-test (*n* = 4, ***p < 0.001). **I** MDA-MB-468 cells with PHGDH knockout (KO#1 and KO#2) via CRISPR/Cas9 were subjected to MTT assays. Four independent experiments were performed and data are shown as the means ± SD with *p*-value based on unpaired *T*-test (*n* = 4, ***p < 0.001). **J**, **K** PHGDH knockout (KO#1 and KO#2) via CRISPR/Cas9 in MDA-MB-468 cells was subjected to colony formation assays. Representative images and quantitative results of colonies are shown. Three independent experiments were performed and data are shown as the means ± SD with *p*-value based on unpaired *T*-test (*n* = 3, **p < 0.01). **L** MDA-MB-468 PHGDH knockout cells were subjected to MTT assay after ASS1 knockout. Four independent experiments were performed and data are shown as the means ± SD with *p*-value based on a two-way ANOVA (*n* = 4, **p < 0.01; n.s., not significant). **M**, **N** MDA-MB-468 PHGDH knockout cells were subjected to a colony formation assay after ASS1 knockout. Representative images and quantitative results of colonies are shown. Three independent experiments were performed and data are shown as the means ± SD with *p*-value based on a two-way ANOVA (*n* = 3, **p < 0.01; n.s., not significant). **O**, **P** MDA-MB-468 cells stably expressing the control empty plasmid or the ASS1 KO, PHGDH KO, ASS1 and PHGDH double knockout plasmids were inoculated subcutaneously into 4 week-old BALB/c nu/nu female mice (*n* = 6). After 42 days of injection, the mice were sacrificed, and the xenograft tumors were removed. Representative tumor volume (**O**) and tumor weight (**P**) are shown. Six independent experiments were performed and data are means ± SD. Significant differences are based on a two-way ANOVA test. (*n* = 6, *p < 0.05, **p < 0.01, n.s. not significant). **Q** Immunoblotting analysis of ASS1 and PHGDH protein expression levels in xenograft tumors. The blots shown are from one representative experiment of three replicates.

Regarding the anticancer effects of ASS1 in TNBC, we also showed that ASS1 knockout in MDA-MB-468 cells significantly promoted cell proliferation (Fig. 5A–C), whereas ASS1 overexpression significantly inhibited cell proliferation. (Fig. 5D–F). Similar results were observed in Hs578T ASS1 knockdown (Fig. 5G) and ASS1 overexpression cells (Fig. 5H). Targeting PHGDH in cancer cells overexpressing PHGDH inhibited cancer cell growth in vitro and in vivo [24, 43, 44]. Thus, we knocked out and knocked down PHGDH, and PHGDH knockout and knockdown significantly inhibited MDA-MB-468 (Fig. 5I–K) and Hs578T cell proliferation (Fig. S8A). Because ASS1 can ubiquitinate and degrade PHGDH, we knocked out or overexpressed ASS1 in PHGDH knockout MDA-MB-468 cells (Fig. S9A, B). PHGDH knockdown abolished the proliferative effect of ASS1 knockdown (Fig. 5L–N) and the inhibition of proliferation by ASS1 overexpression (Fig. S10A–C) in the same experiment. We also examined whether the negative regulation of PHGDH contributes to the anticancer effects of ASS1 in vivo by using xenografts of TNBC in nude mice. Compared to control cells, ASS1 knockout greatly enhanced the growth of xenograft breast tumors, whereas PHGDH knockout largely inhibited the growth of xenograft breast tumors (Fig. 5O). Interestingly, the effect of ASS1 knockout to promote tumor growth was largely abrogated by PHGDH knockout. Furthermore, the weight of breast tumor xenografts in derived from ASS1 knockout cells significantly increased, but the weight of breast tumor xenografts derived from PHGDH knockout cells decreased greatly relative to that of control cells. Similarly, the increase in tumor weight induced by ASS1 knockout was largely abrogated by further knockout of PHGDH (Fig. 5P). Moreover, we further determined the protein expression of the tumor by western blot analysis. Consistent with the previous results, ASS1 knockout also obviously increased the protein level of PHGDH in vivo (Fig. 5Q). Collectively, the above results demonstrated that the downregulation of PHGDH by ASS1 contributes significantly to the anticancer effects of ASS1.

### ASS1 inhibits TNBC growth by attenuating PHGDH mediated serine synthesis

**Fig. 6:**
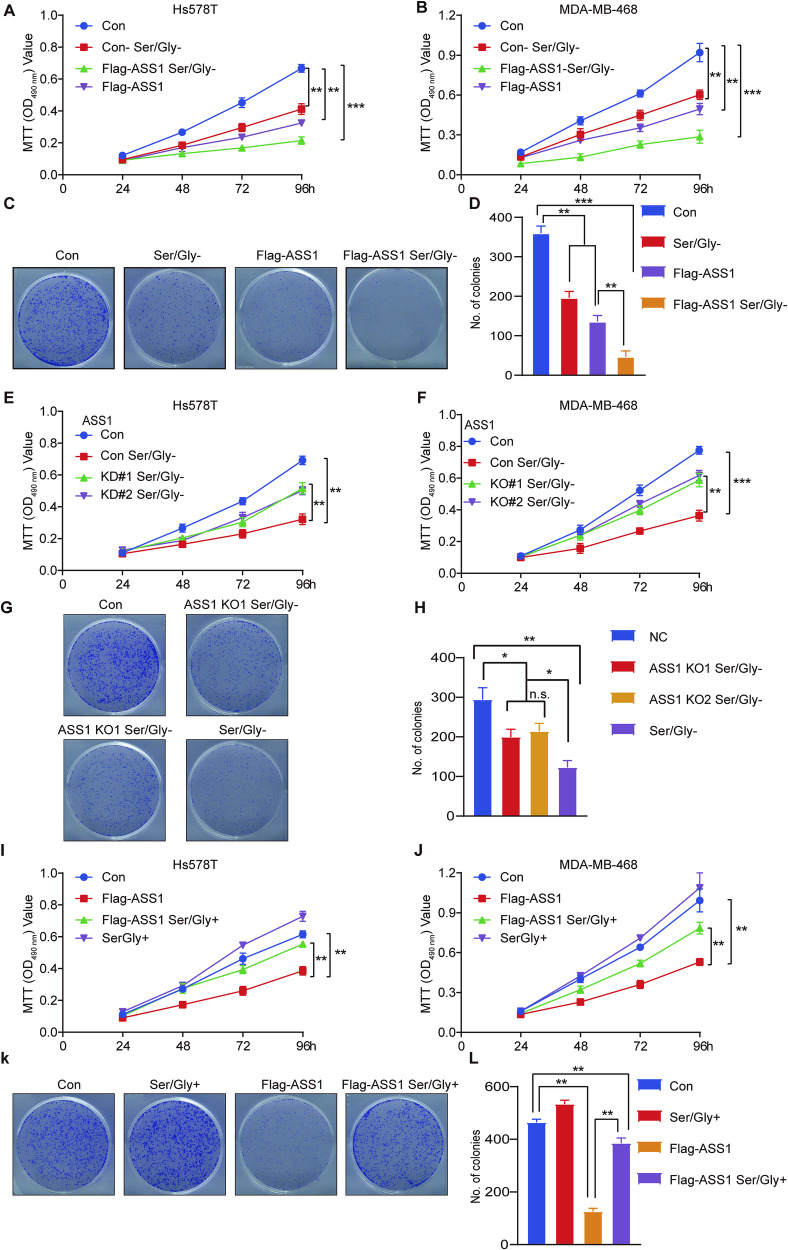
ASS1 inhibits the proliferation of TNBC cells mainly by inhibiting serine synthesis. **A** Hs578T cells stably expressing the control empty plasmid or Flag-ASS1, were deprived or not of serine and glycine and subjected to MTT assays. Four independent experiments were performed and data are shown as the means ± SD with *p-*value based on two-way ANOVA (*n* = 4, **p < 0.01, ***p < 0.001). **B** MDA-MB-468 cells stably expressing the control empty plasmid or Flag-ASS1, were deprived or not of serine and glycine and subjected to MTT assays. Four independent experiments were performed and data are shown as the means ± SD with *p-*value based on two-way ANOVA (*n* = 4, **p < 0.01, ***p < 0.001). **C**, **D** MDA-MB-468 cells stably expressing the control empty plasmid or Flag-ASS1, were deprived or not of serine and glycine and subjected to colony formation assays. Representative images and quantitative results of colonies are shown. Three independent experiments were performed and data are shown as the means ± SD with *p-*value based on a two-way ANOVA (*n* = 3, **p < 0.01, n.s., not significant). **E** Hs578T cells with or without depletion of serine and glycine and ASS1 knockdown cells (KD#1 and KD#2) depleted of serine and glycine were subjected to MTT assays. Four independent experiments were performed and data are shown as the means ± SD with *p*-value based on two-way ANOVA (*n* = 4, *p < 0.05, **p < 0.01). **F** MDA-MB-468 cells with or without depletion of serine and glycine and ASS1 knockout cells (KO#1 and KO#2) depleted of serine and glycine were subjected to MTT assays. Four independent experiments were performed and data are shown as the means ± SD with *p-*value based on two-way ANOVA (*n* = 4, **p < 0.01, ***p < 0.001). **G**, **H** MDA-MB-468 cells with or without depletion of serine and glycine and ASS1 knockout cells (KO#1 and KO#2) depleted of serine and glycine were subjected to colony formation assays. Representative images and quantified results of colonies are shown. Three independent experiments were performed and data are shown as the means ± SD with *p*-value based on two-way ANOVA (*n* = 3, *p < 0.05, **p < 0.01, n.s., not significant). **I** Hs578T cells stably expressing the control empty plasmid or Flag-ASS1 supplemented with or without 10 mM serine and glycine in normal media were subjected to MTT assays. Four independent experiments were performed and data are shown as the means ± SD with *p*-value based on a two-way ANOVA (*n* = 4, **p < 0.01). **J** MDA-MB-468 cells stably expressing the control empty plasmid or Flag-ASS1 supplemented with or without 10 mM serine and glycine in normal media were subjected to MTT assays. Four independent experiments were performed and data are shown as the means ± SD with *p-*value based on two-way ANOVA-test (*n* = 4, **p < 0.01). **K**, **L** MDA-MB-468 cells stably expressing the control empty plasmid or Flag-ASS1 supplemented with or without 10 mM serine and glycine in normal media were subjected to colony formation assays. Representative images and quantified results are shown. Three independent experiments were performed and data are shown as the means ± SD with *p*-value based on a two-way ANOVA (*n* = 3, **p < 0.01).

Previous results showed that ASS1 overexpression inhibited serine synthesis. Here, we explored whether ASS1 inhibits TNBC cell proliferation by inhibiting serine synthesis. We found that ASS1 overexpression showed a more potent repressive effect on the proliferation of Hs578T (Fig. 6A) and MDA-MB-468 cells (Fig. 6B–D) under serine- and glycine-depleted conditions. In addition, PHGDH knockout and knockdown also had more significant inhibitory effects on the two cell lines, and ASS1 knockout or knockdown showed no significant promoting effect on PHGDH knockout or knockdown cells (Fig. S11A, B). Consistent with a previous study [24], our results also showed that the proliferation of cells with elevated PHGDH was inhibited in serine- and glycine-deficient medium. Since ASS1 knockout and knockdown promoted serine synthesis, we determined whether ASS1 knockout and knockdown can rescue the inhibitory effect of serine and glycine depletion. Interestingly, we found that ASS1 knockout and knockdown promoted Hs578T (Fig. 6E) and MDA-MB-468 (Fig. 6F–H) cell proliferation in serine- and glycine-deficient medium. Conversely, cell proliferation inhibited by ASS1 overexpression was rescued by excess serine and glycine (Fig. 6I–L). Together, the above results demonstrated that ASS1 affects TNBC cell proliferation mainly through serine synthesis and that overexpression of ASS1 or knockdown or knockdown of PHGDH renders cells dependent on extracellular serine and glycine.

## Discussion

**Fig. 7:**
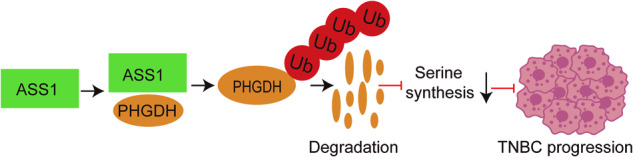
Mechanistic model in which ASS1 suppresses serine synthesis and TNBC progression via PHGDH ubiquitination and degradation. ASS1 directly interacts with the PHGDH protein, leading to its degradation through the proteasome, subsequently inhibiting serine synthesis and ultimately the progression of TNBC.

ASS1 is frequently expressed at low levels in numerous cancers [5, 6]. In our earlier research, we discovered that activating ASS1 with an activator can suppress cancer growth by decreasing pyrimidine synthesis. However, the fact that adding pyrimidine only partially restored cell viability indicates the possibility of an alternative antitumor pathway involving ASS1 [13], and the signaling pathways and interacting proteins of ASS1 in cancer have not been fully elucidated. Here, we showed that PHGDH is an important protein that interacts with ASS1 and contributes to the anticancer effects of ASS1 in human TNBC by degrading PHGDH to inhibit serine synthesis and tumorigenesis (Fig. 7). ASS1 knockout and knockdown promote PHGDH protein stabilization to activate serine synthesis and promote cell proliferation and, consequently, tumorigenesis, which can be greatly abrogated by PHGDH knockout. These findings provide powerful evidence that the distinctive mechanism by which ASS1 exerts its anticancer effects involves the downregulation of PHGDH and serine synthesis.

Our study indicated that PHGDH is an important protein that interacts with ASS1 and further determined that the catalytic domain of PHGDH directly interacts with ASS1. However, the exact binding site needs to be further explored. We also found that changes in ASS1 increased or decreased PHGDH protein levels, whereas knockout or knockdown of PHGDH did not affect ASS1 expression, suggesting that ASS1 may act as an upstream regulator. Furthermore, ASS1 promoted the ubiquitination-mediated of PHGDH. However, ASS1 is a urea cycle enzyme that catalyzes arginine synthesis. Therefore, we speculate that deubiquitination enzymes or E3 ubiquitin ligases may be present in and activated by ASS1 to degrade PHGDH. Exploring this mechanism will be interesting in future work. In addition, our IHC results showed a negative correlation between ASS1 and PHGDH protein expression in clinical samples of recurrent and nonrecurrent TNBC patients, which indicates that high PHGDH and low ASS1 expression may be associated with TNBC recurrence.

Serine and glycine, two nonessential amino acids in mammals, are critical for cell proliferation [45–47]. Since ASS1 degrades PHGDH, overexpression of ASS1 inhibited serine synthesis, and knockout of PHGDH abrogated this effect, which similar to the findings of Liu et al. [39]. Moreover, the changes in serine and glycine levels caused by ASS1 were largely abolished by PHGDH knockout and knockdown. Our results also showed that ASS1 knockout and knockdown promote TNBC cell proliferation in vitro and in vivo; however, ASS1 overexpression suppressed cell proliferation, which is consistent with a recent study showing that ASS1 knockdown promotes while ASS1 overexpression inhibits MDA-MB-231 cell growth and tumor development [13]. Moreover, PHGDH knockout abolished the proliferation-promoting effect of ASS1 knockout in vitro and in vivo, which indicated that the tumor suppressor function of ASS1 greatly depends on PHGDH. PHGDH knockdown and its small-molecule inhibitors are selectively toxic to PHGDH-dependent cells and tumors, even in the presence of serine [43]. Our results also showed that PHGDH knockout or knockdown and ASS1 overexpression significantly inhibited the proliferation of MDA-MB-468 and Hs578T cells.

Several studies have demonstrated that cells with high levels of the PHGDH protein are also capable of proliferating under serine- and glycine-depleted conditions, whereas in cells with low levels of PHGDH, deprivation of serine and glycine in the medium strongly inhibits proliferation [24, 39]. In our study, we found that ASS1 overexpression, PHGDH knockout or knockdown resulted in significant inhibition or even cessation of proliferation in cells overexpressing PHGDH when serine and glycine were removed from the medium. We speculate that the decrease in the PHGDH protein may have made it sensitive to the presence or absence of extracellular serine and glycine. Moreover, ASS1 knockout and knockdown partially rescued cell proliferation in serine- and glycine- derived media. Importantly, excess serine and glycine largely reversed the inhibition of proliferation caused by ASS1 overexpression, which is consistent with the findings of the pancreatic cancer study by Ma et al. [30]. Although cells with elevated PHGDH are dependent mainly on serine synthesis, external amino acids are also important for cell proliferation. Inhibitors of PHGDH, such as NCT-503 and CBR-5884, have been shown to suppress cell proliferation and tumor development by targeting PHGDH [43, 44]. Novel PHGDH allosteric inhibitors also displayed robust inhibitory effects on cell lines with PHGDH amplified in vitro and in vivo [48]. We speculate that these inhibitors combined with serine- and glycine-deficient conditions may also play a more significant role in inhibiting cancer development and progression.

In summary, we identified PHGDH as an interacting protein of ASS1. ASS1 suppresses serine synthesis via ubiquitination and degradation of PHGDH, which contributes significantly to the anticancer effects of ASS1. ASS1 knockout or knockdown enhanced PHGDH protein stabilization to increase serine synthesis and promote cancer cell proliferation and, consequently tumorigenesis, providing a novel anticancer mechanism of ASS1 in TNBC.

### Supplementary information

Supplementary materials

Original Data File

## Data Availability

All cell lines, plasmids, datasets, and other stable reagents generated in this study are available from the corresponding author with a reasonable request.
