# Supplementary material for: ASS1 inhibits triple-negative breast cancer by regulating PHGDH stability and de novo serine synthesis
Source: Cell Death Dis. 2024 May 6;15(5):319. doi: 10.1038/s41419-024-06672-z (PMC11074131; doi:10.1038/s41419-024-06672-z)
Supplement: Supplementary file 1 — Supplementary materials [file 41419_2024_6672_MOESM1_ESM.docx]

**Supplementary materials and methods**

**Full title:** **ASS1 inhibits triple-negative breast cancer by regulating PHGDH stability and *de novo* serine synthesis**

**Table S1. The oligonucleotides** **used for qRT‒PCR (5’-3’) are as follows:**

| GAPDH forward | CGACCACTTTGTCAAGCTCA |
| --- | --- |
| GAPDH reverse | GGGTCTTACTCCTTGGAGGC |
| ASS1 forward | GAGGATGCCTGAATTCTACA |
| ASS1 reverse | GTTGGTCACCTTCACAGG |
| PHGDH forward | ATCTCTCACGGGGGTTGTG |
| PHGDH reverse | AGGCTCGCATCAGTGTCC |

**Table S2. primary and secondary antibody information used in this study**

| **Antibodies** | **Source** | **Catalog#** | **Dilution** | **Application** |
| --- | --- | --- | --- | --- |
| ASS1 | Cell Signaling | 70720 | 1:1000; 1:250  1:100; 1:1600 | WB; IHC  IP; IF |
| PHGDH | Cell Signaling | 66350 | 1:1000; 1:50 | WB; IP |
| PHGDH | Proteintech | 14719-1-AP | 1:500 | IHC |
| PHGDH | Proteintech | 67591-1-Ig | 1:400 | IF |
| GAPDH | Abbkine | ABP50163 | 1:10000 | WB |
| HA-tag | Cell Signaling | 3724 | 1:1000 | WB |
| LC3B | Cell Signaling | 3868 | 1:1000 | WB |
| Ub | Cell Signaling | 3936 | 1:1000 | WB |
| GST-tag | Abbkine | ABM40020 | 1:5000 | WB |
| Flag-tag | ABclonal | AE005 | 1:100 | IP |
| Flag-tag | ABclonal | AE063 | 1:2000 | WB |
| c-Myc-tag | AFFINITY | AF6054 | 1:1000 | WB |
| Normal Rabbit IgG | Cell Signaling | 2729 | 1:100 | IP |
| IPKine™ HRP Mouse Anti-Rabbit IgG LCS | Abbkine | A25022 | 1:2000 | WB |
| Goat Anti-Rabbit IgG-CY3 | jackson immuno research | 11-167-003 | 1:400 | IF |
| Goat Anti-Rabbit IgG | Abbkine | A21020 | 1:10000 | WB |
| Goat Anti-Mouse IgG | Abbkine | A21010 | 1:10000 | WB |
| CoraLite488 – conjugated Affinipure Goat Anti-Mouse IgG(H+L) | Proteintech | SA00013-1 | 1:500 | IF |

**Table S3. The oligonucleotides** **used for sgRNA (5’-3’) are as follows:**

| ASS1sgRNA1 forward | CACCGCAGCCACACGAGGATGCACG |
| --- | --- |
| ASS1sgRNA1 reverse | AAACCGTGCATCCTCGTGTGGCTGC |
| ASS1sgRNA2 forward | CACCGCGAGGATGCACGAGGTGTCC |
| ASS1sgRNA2 reverse | AAACGGACACCTCGTGCATCCTCGC |
| PHGDH sgRNA1 forward | CACCGTGCAAGATCTTCCGGCAGCA |
| PHGDH sgRNA1 reverse | AAACTGCTGCCGGAAGATCTTGCAC |
| PHGDH sgRNA2 forward | CACCG TGCCGGAAGATCTTGCAAGA |
| PHGDH sgRNA2 reverse | AAACTCTTGCAAGATCTTCCGGCAC |

**Table S4. The oligonucleotides** **used for shRNA (5’-3’) are as follows:**

| ASS1shRNA1 | CCGGGCTGAAGGAACAAGGCTATGACTCGAGTCATAGCCTTGTTCCTTCAGCTTTTTG |
| --- | --- |
| ASS1 shRNA2 | CCGGGCTATGACGTCATTGCCTATCCTCGAGGATAGGCAATGACGTCATAGCTTTTTG |
| PHGDHshRNA1 | CCGGCTTAGCAAAGAGGAGCTGATACTCGAGTATCAGCTCCTCTTTGCTAAGTTTTTG |
| PHGDH shRNA2 | CCGGCAGACTTCACTGGTGTCAGATCTCGAGATCTGACACCAGTGAAGTCTGTTTTTG |

**Figure S1. Purification of His-PHGDH and His-ASS1protein.**


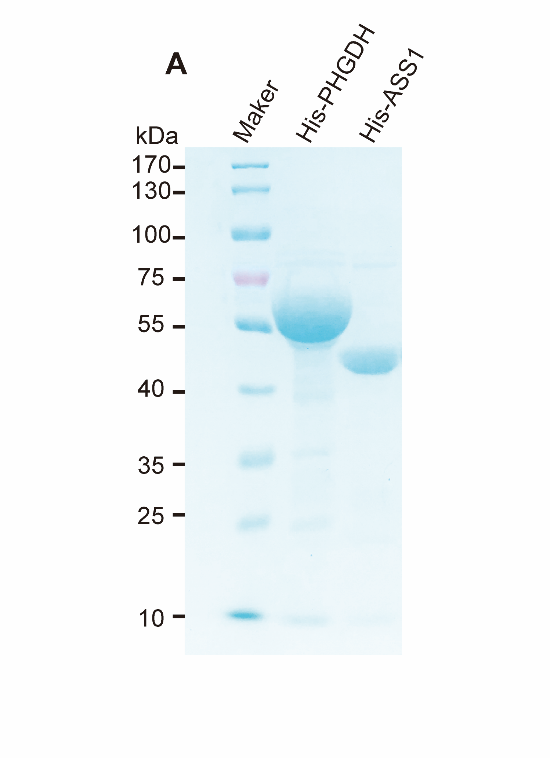


(A) His-PHGDH and His-ASS1 proteins were purified to homogeneity by immobilized metal affinity chromatography (Ni^2+^) and analyzed by SDS-PAGE.

**Figure S2. Unique peptides of PHGDH identified by LC‒MS/MS.**

(A) The position of the specific peptide identified by LC‒MS/MS in the amino acid sequence of PHGDH was determined by sequence comparison. The peptide sequences marked in red are those identified by LC‒MS/MS, ten peptides in total. Two specific peptides are distinguished by the yellow background.

**Figure S3. ASS1 protein expression levels were not affected by PHGDH.**


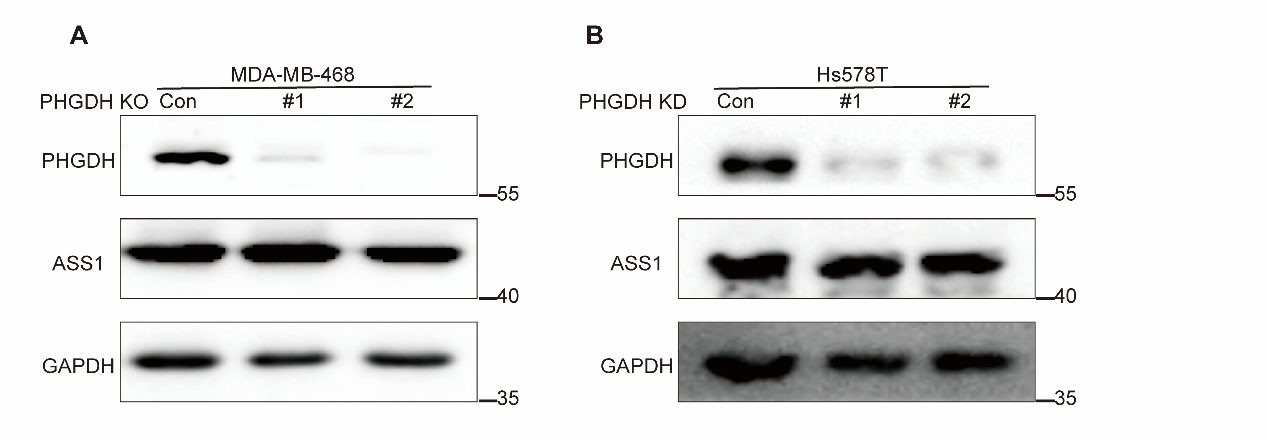


(A-B) Immunoblotting assays to determine the expression levels of ASS1 with PHGDH knockout (A) and PHGDH knockdown(B) in MDA-MB-468 and Hs578T cells, respectively.

**Figure S4. PHGDH mRNA expression levels were not affected by ASS1.**


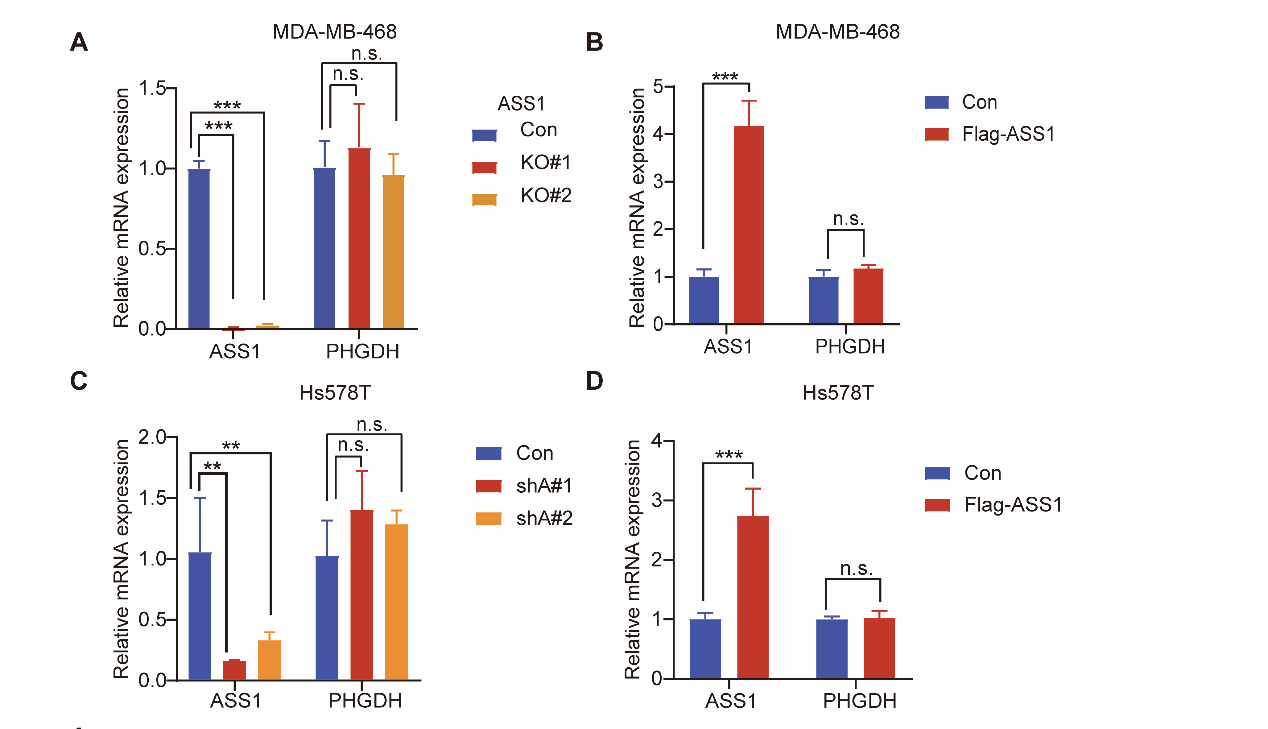


(A-B) qRT‒PCR analysis of ASS1 and PHGDH levels in MDA-MB-468 cells with ASS1 knockout(A) and overexpression(B). Three independent experiments were performed and data are shown as the mean ± SD with p value based on two-way ANOVA. (n=3, **p < 0.01, ***p < 0.001, n.s., not significant).

(C-D) qRT‒PCR analysis of ASS1 and PHGDH levels in HS578T cells with ASS1 knockdown(C) and overexpression(D). Three independent experiments were performed and data are shown as the mean ± SD with p value based on two-way ANOVA. (n=3, **p < 0.01, ***p < 0.001, n.s., not significant).

**Figure S5. ASS1 and PHGDH are negatively correlated in non-recurrent TNBC tissues.**


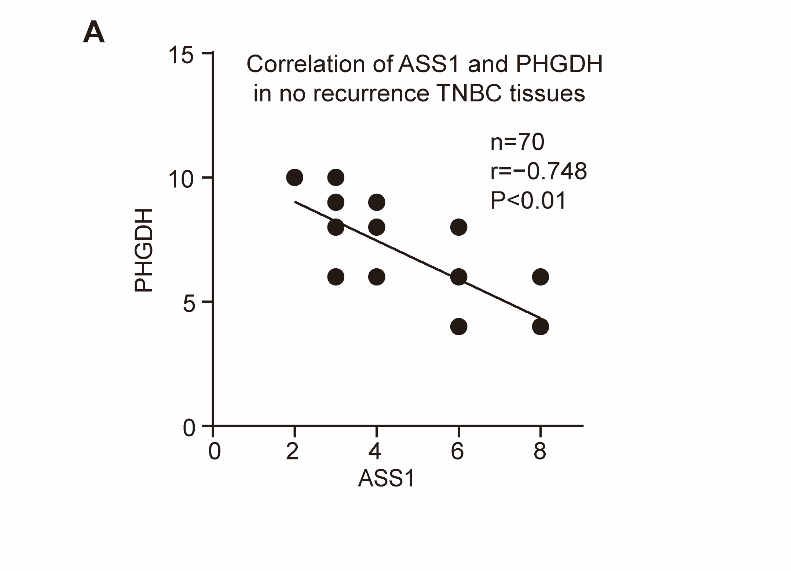


1. Correlation analysis of ASS1 and PHGDH protein expression in human no recurrent triple-negative breast cancer tissues (n=70) revealed a significant correlation (Pearson's correlation, r= −0.748; p < 0.01).

**Figure S6. PHGDH levels in cells were not affected by BafA1.**


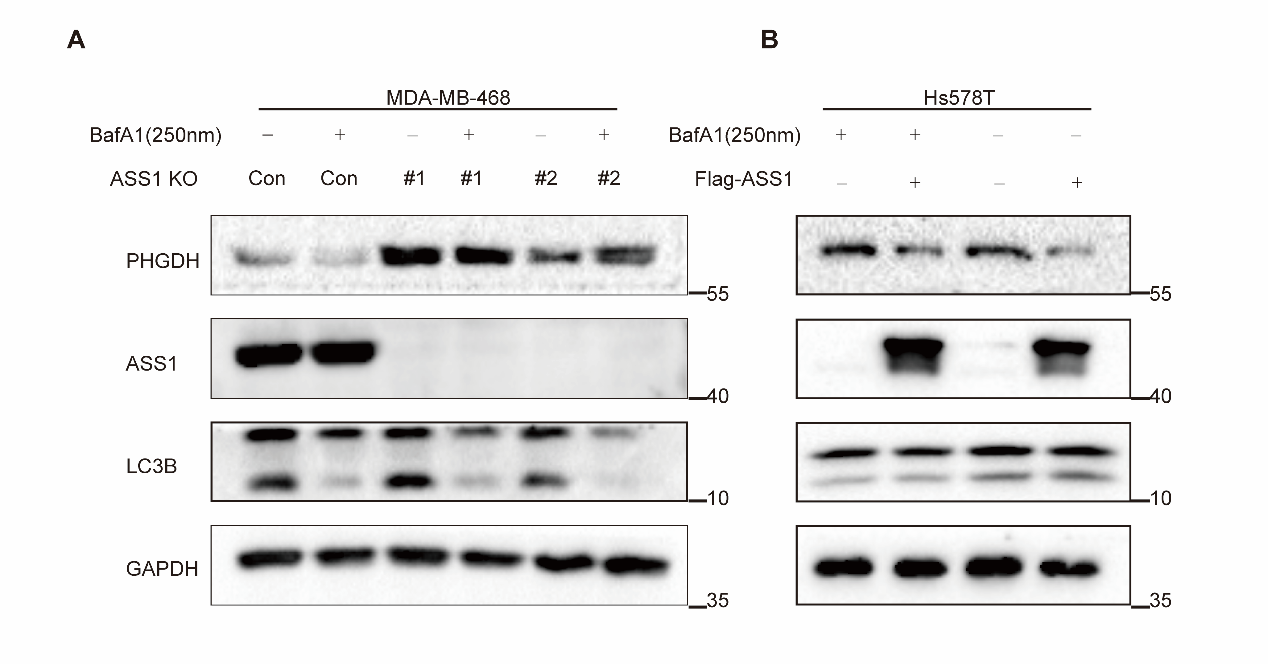


(A) Immunoblotting assays examining the expression levels of PHGDH in MDA-MB-468 cells of ASS1 knockout treated with DMSO or 250nM BafA1 for 8 hours.

(B) Immunoblotting assays examining the expression levels of PHGDH in Hs578T cells of ASS1 overexpression treated with DMSO or 250nM BafA1 for 8 hours.

**Figure S7. Flag-ASS1 expression inhibits serine synthesis through PHGDH.**


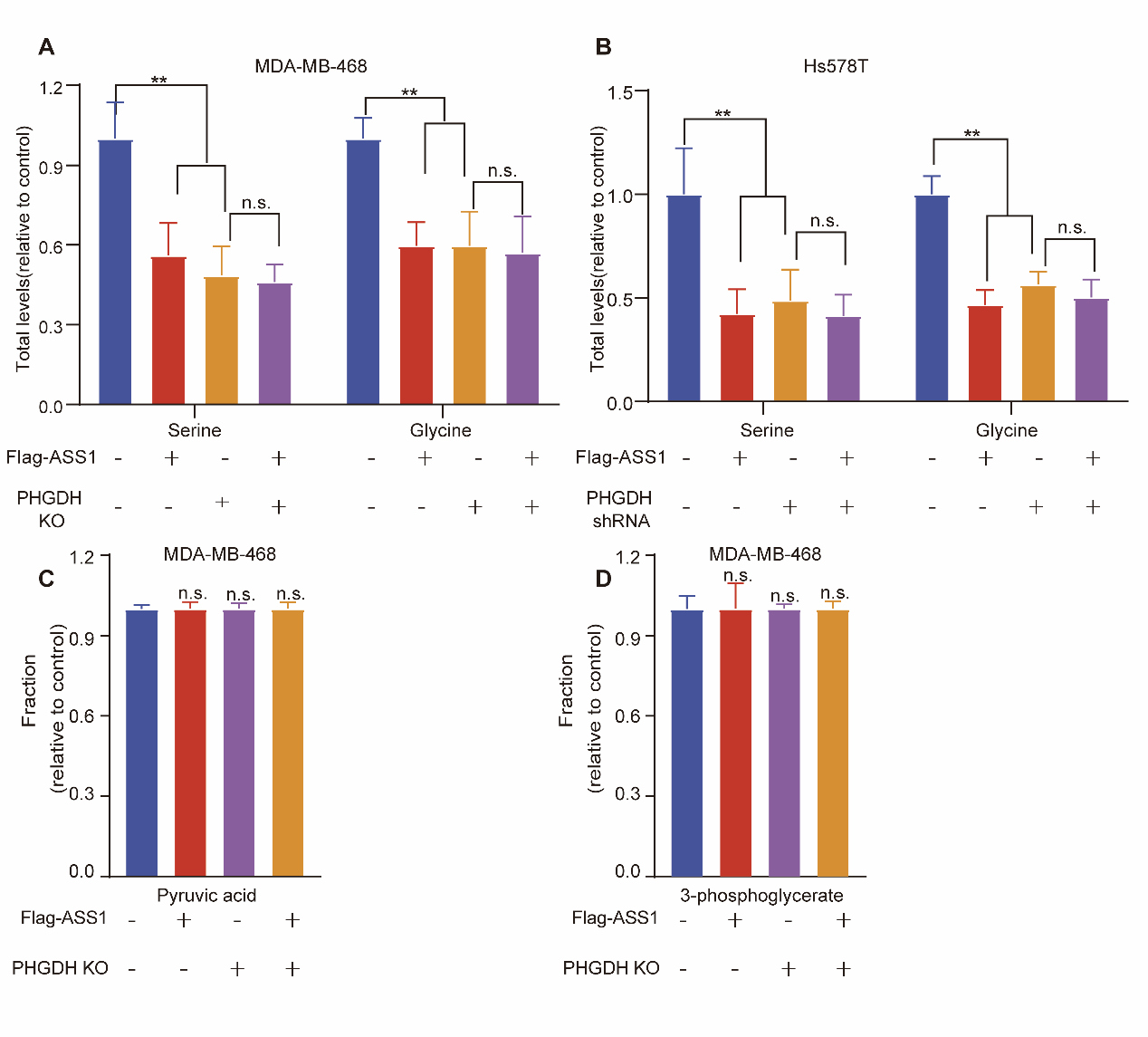


(A) MDA-MB-468 cells overexpressing ASS1 were knocked out PHGDH by CRISPR/Cas9 and subjected to LC-MS/MS to determine the concentrations of serine and glycine levels. Six independent experiments were performed and data are means ± SD with p values based on two-way ANOVA test. (n=6, *p <0.05, **p < 0.01, n.s., not significant).

(B) Hs578T cells overexpressing ASS1 were knocked down PHGDH by shRNA and subjected to LC-MS/MS to determine the concentrations of serine and glycine levels. Six independent experiments were performed and data are means ± SD with p values based on two-way ANOVA test. (n=6, *p <0.05, **p < 0.01, n.s., not significant).

(C-D) MDA-MB-468 cells cultured in medium containing 25 mM [U-13C] D-glucose with 0.4mM serine/glycine were lysed for stable isotope flux analysis. Neither Flag-ASS1, PHGDH knockout nor Flag-ASS1 or PHGDH knockout significantly affected the incorporation of ^13^C into pyruvic acid(C) and 3-phosphoglycerate(D) in MDA-MB-468 cells. Three independent experiments were performed and data are the means ± SD with p values based on one-way ANOVA. (n=3, n.s., not significant).

**Figure S8. Knockdown of PHGDH in Hs578T cells inhibits cell proliferation.**


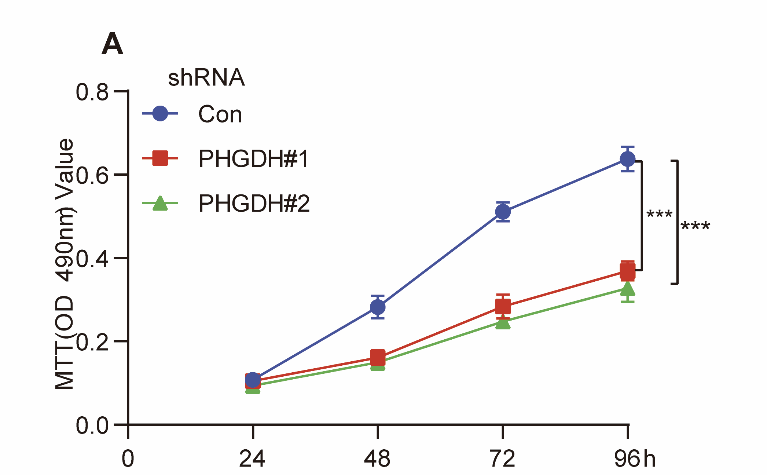


(A) Hs578T cells with PHGDH knockdown (KD#1 and KD#2) via shRNA were subjected to MTT assays. Representative images of the survival curves are shown. Four independent experiments were performed and data are shown as the mean ± SD with p value based on a two-way ANOVA (n=4, **p < 0.01, ***p < 0.001, n.s., not significant).

**Figure S9. Western blot results in PHGDH knockout MDA-MB-468 cells that were treated with ASS1 overexpression and ASS1 knockout.**


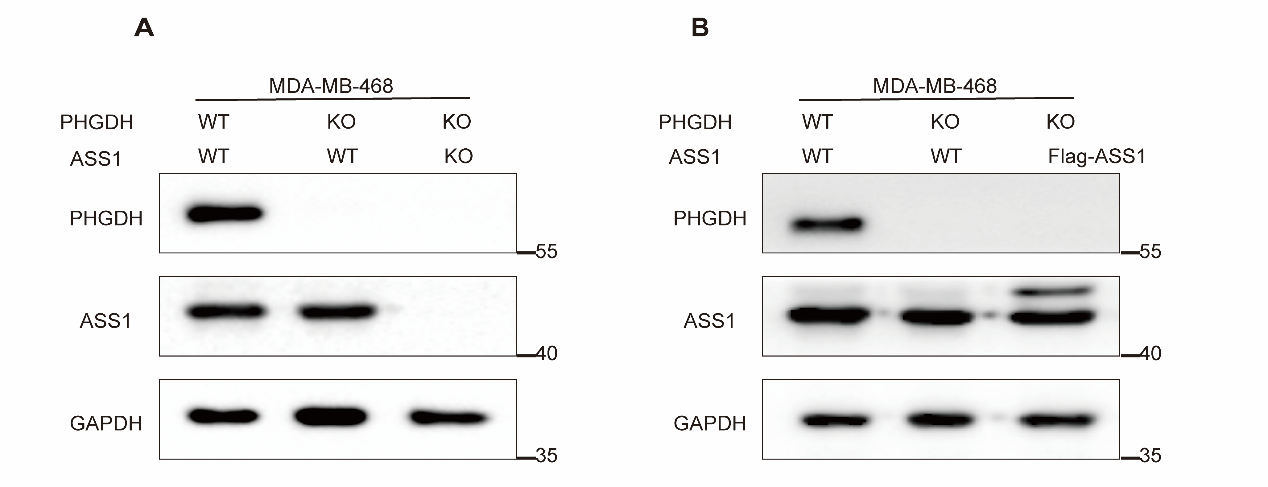


(A, B) Immunoblotting assays to determine the expression levels of PHGDH in PHGDH knockout MDA-MB-468 cells that were treated with ASS1 knockout(A) and ASS1 overexpression (B).


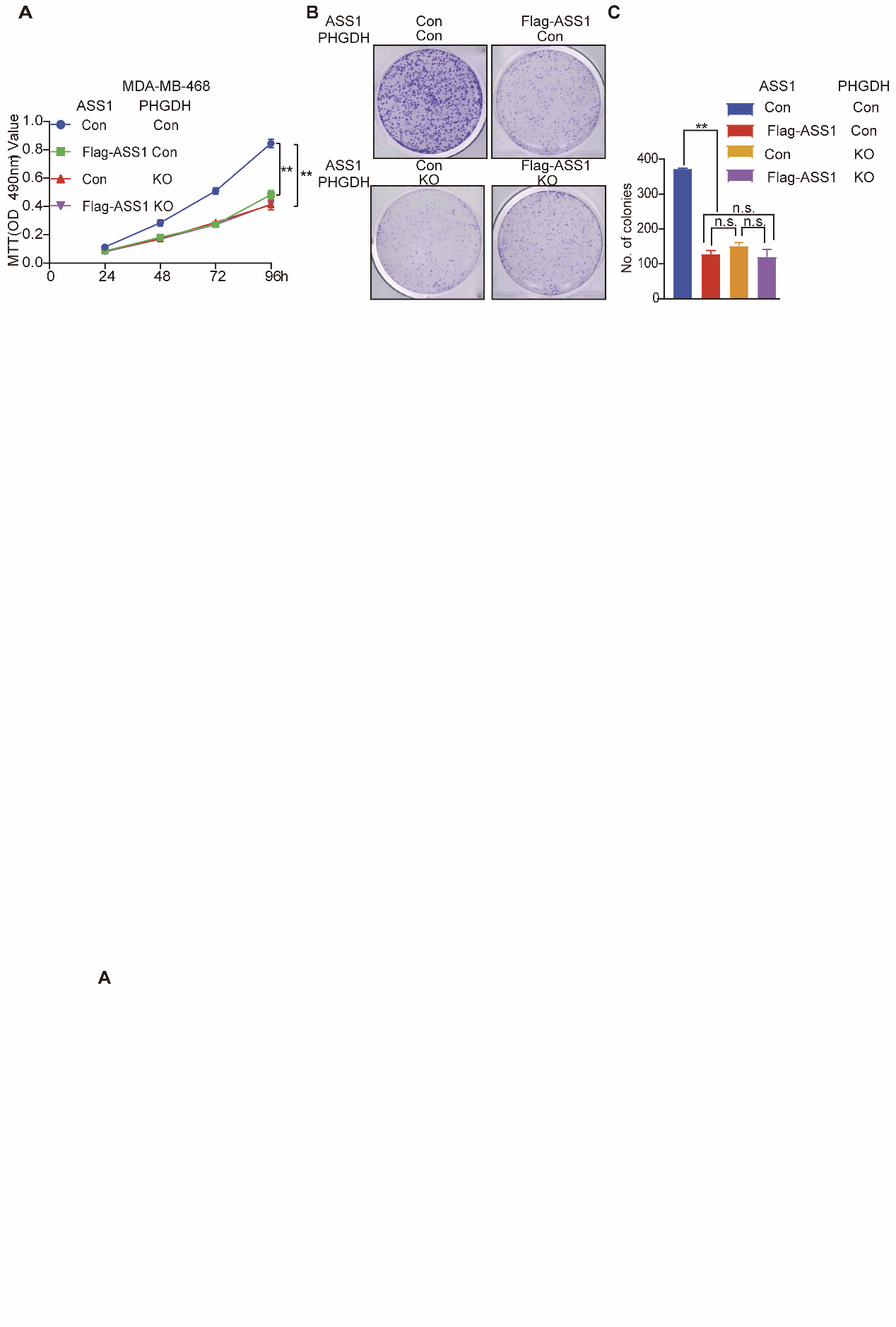
**Figure S10. PHGDH knockout abrogates the proliferation inhibition of ASS1 overexpression.**

(A) PHGDH knockout MDA-MB-468 cells were subjected to MTT assays after ASS1 overexpression. Four independent experiments were performed and data are shown as the mean ± SD with p value based on two-way ANOVA (n=4, **p < 0.01, n.s., not significant).

(B-C) PHGDH knockout MDA-MB-468 cells were subjected to colony formation assays after ASS1 overexpression. Representative images and quantitative results of colonies are shown. Three independent experiments were performed and data are shown as the mean ± SD with p value based on two-way ANOVA (n=3, **p < 0.01, n.s., not significant).

**Figure S11. Serine and glycine deprivation enhance the effect of PHGDH knockdown in inhibiting breast cancer cell proliferation.**


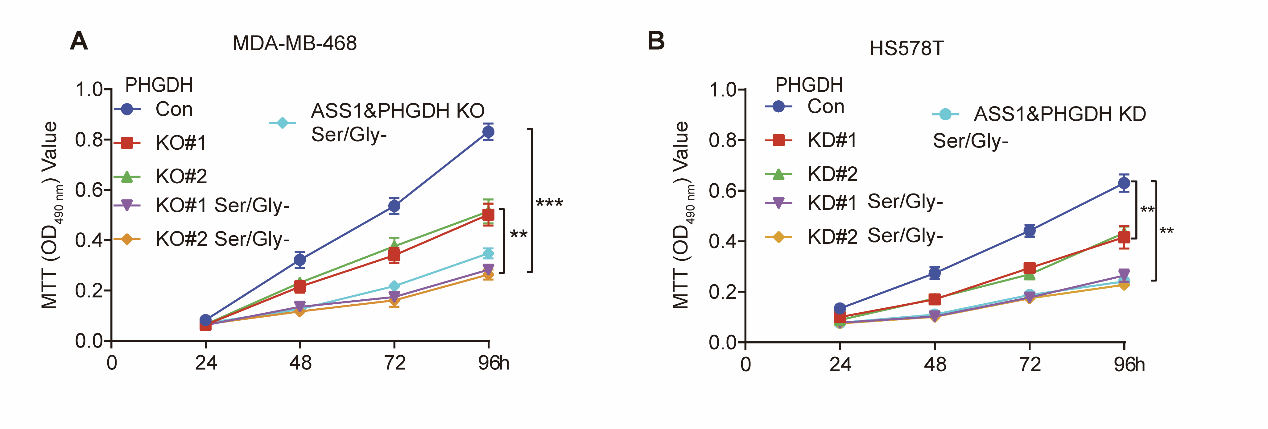


**(A)** PHGDH knockout (KO#1 and KO#2), PHGDH and ASS1 knockout by CRISPR/Cas9 in MDA-MB-468 cells deprived or not of serine and glycine were subjected to MTT assays. Representative images of the survival curves are shown. Four independent experiments were performed and data are shown as the mean ± SD with p value based on two-way ANOVA. (n=4, **p < 0.01, ***p < 0.001, n.s., not significant).

**(B)** PHGDH knockdown (KD#1 and KD#2), PHGDH and ASS1 knockdown by shRNA in Hs578T cells deprived or not of serine and glycine were subjected to MTT assays. Representative images of the survival curves are shown.

Four independent experiments were performed and data are shown as the mean ± SD with p value based on two-way ANOVA. (n=4, **p < 0.01).

**Data processing and instrument parameter settings of LC-MS/MS.** The LC-MS/MS is equipped with AB SCIEXQTRAP®6500+LC-MS/MS system and an electrospray ion source operating in anion mode for the analysis of serine and glycine concentrations. Data were acquired and analyzed using Analyst (version 1.6.3, AB) and MultiQuant (version 3.0.2, AB) software, respectively. C18 100A UPLC Kinetex® column has an internal diameter of 100 mm×2.1 mm and a length of 1.7 μm. The mobile phase wad 5 M ammonium formate/acetonitrile=99.1/0.9. The flow rate was 0.2ml/min. The column temperature was 40°C. The sample volume was 1 µL and the temperature was maintained at 8°C.

The mass spectrum uses nitrogen as the collision gas, the curtain gas is 35.0 psi, and the collision gas is medium. The electron spray voltage is -4.5 kV, the temperature is 450°C, the spray gas is 55.0 psi, the impact voltage is -16000 V, the declustering voltage is -60000 V, the input voltage is -10000 V, the impact pool output voltage is -1500 V, and the voltage hold time is 130.0ns.


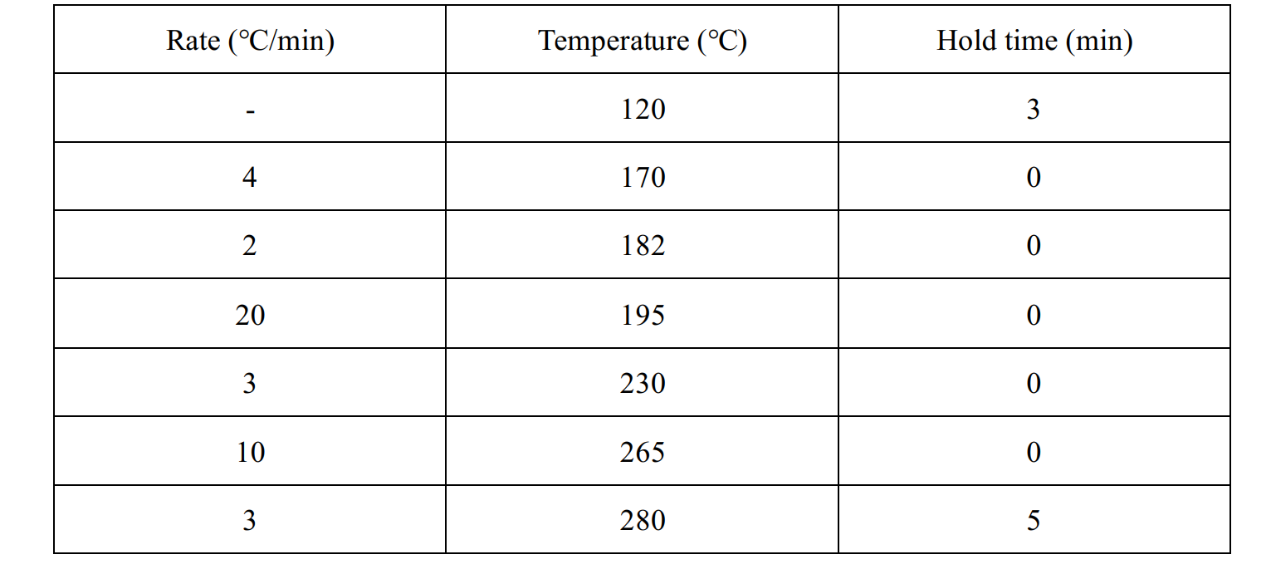
**GC-MS analysis and data Analysis.** A Shimadzu QP-2020 GC-MS was programmed with an injection temperature of 250°C injection and injected with 5 µl of sample.The gradient elution of GC oven temperature was list as follows:

GC flow rate with helium carrier gas was 0.92 ml/min. The GC column used was a 30 m x 0.25mm x 0.25 mm DB-5ms. GC-MS interface temperature was 300°C and (electron impact) ion source temperature was set at 200 °C, with 70 V ionization voltage. The mass spectrometer was set to scan m/z range 50-700, with 1 kV detector.

GC/MS data were analyzed to determine isotope labeling and quantities of metabolites. To determine 13C labeling, the mass distribution for known fragments of metabolites was extracted from the appropriate chromatographic peak. These fragments contained either the whole carbon skeleton of the metabolite, or lacked the alpha carboxyl carbon, or (for some amino acids) contained only the backbone minus the side-chain^1^ . For each fragment, the retrieved data comprised mass intensities for the lightest isotopomer (without any heavy isotopes, M0), and isotopomers with increasing unit mass (up to M6) relative to M0. These mass distributions were normalized by dividing by the sum of M0 to M6 and corrected for the natural abundance of heavy isotopes of the elements H, N, O, Si and C, using matrix-based probabilistic methods as described^2^, and implemented in code.

**IHC score.** IHC staining for ASS1 and PHGDH was calculated using semi-quantitative scoring criteria for staining intensity and positive cell frequency. Staining intensity was defined as no staining, weak staining, moderate staining and strong staining depending on the stained sample. Positive cell frequency was defined as 0-5%, <5-25%, 25%-50%, 50%-75%, 75%-100% and recorded as 0, 1, 2, 3, 4. The immunoreactivity score is equal to the staining intensity multiplied by the frequency of positive cells.

**References**

1. van Winden WA, Wittmann C, Heinzle E, Heijnen JJ. Correcting mass isotopomer distributions for naturally occurring isotopes. Biotechnol Bioeng 2002, 80(4): 477-479.

2. Portnoy VA, Scott DA, Lewis NE, Tarasova Y, Osterman AL, Palsson BØ. Deletion of genes encoding cytochrome oxidases and quinol monooxygenase blocks the aerobic-anaerobic shift in Escherichia coli K-12 MG1655. Appl Environ Microbiol 2010, 76(19): 6529-6540.
